# 

**Published:** 1883-03

**Authors:** 


### CONTENTS:

ART. I.—Attachment of Artificial Crowns to Natural Roots. By H. W. F. Buttner, D. D. S. .......481

ART. II.—Relaxation for Dentists. By W. C. Barrett, D D. S. .......494

ART. III.—Natural Abilities and Mental Requirements in the Study and Practice of Dentistry. By Dr. Morrell A. Webb. .......500

ART. IV.—The Study of the Face as an Index of the Brain. By Dr. F. Warner. .......504

ART. V.—Marshall H. Webb. D. D. S. By H. C. Longnecker, D D. S. .......509

ART. VI.—Pulp Nodules and Their Treatment. By H. L Gilmour, D. D. S. .......513

ART. VII.—Plaster of Paris vs. Modeling Composition. By Dr. Stewart I. Spence. .......515

ART. VIII.—Uniting Gold to Amalgam in Tooth Filling. By N. W. Kingsley, D. D. S. .......517

ART. IX.—A Case of Gangrena Oris. B D. A. Hengst, M. D. .......519

Death of Dr. Wm. H. Allen .......521

### EDITORIAL, ETC.

The Clinics at the Dental Department of the University of Maryland .......522
Nitrous Oxide Gas .......523
Seventy-Sixth Annual Commencement of the University of Maryland .......524
Ether Spray as a Cure for Neuralgia .......524

### MONTHLY SUMMARY.

The Use of Tobacco by Children .......525
An Idea on Amalgams .......526
Carbolic Acid Poisoning .......527
A New Hypnotic and Depresso-Motor .......528
Influence of Electric Light on Health .......528

